# Triglyceride-glucose index trajectory and stroke incidence in patients with hypertension: a prospective cohort study

**DOI:** 10.1186/s12933-022-01577-7

**Published:** 2022-07-27

**Authors:** Zegui Huang, Xiong Ding, Qing Yue, Xianxuan Wang, Zekai Chen, Zefeng Cai, Weijian Li, Zhiwei Cai, Guanzhi Chen, Yulong Lan, Weiqiang Wu, Shouling Wu, Youren Chen

**Affiliations:** 1 Shantou University Medical College, Shantou, China; 2 Department of Cardiology, Second Affiliated Hospital of Shantou University Medical College, 69 Dongxia North Road, Shantou, 515000 China; 3 School of Public Health, Wuhan University, Wuhan, China; 4 School of Public Health, North China University of Science and Technology, Tangshan, China; 5 Department of Epidemiology, University Medical Center Groningen, University of Groningen, Groningen, the Netherlands; 6 China Medical University, Shenyang, China; 7 Department of Cardiology, Kailuan General Hospital, 57 Xinhua East Road, Tangshan, 063000 China

**Keywords:** Triglyceride-glucose index, Trajectory, Insulin resistance, Stroke, Hypertension

## Abstract

**Background:**

It has been suggested that the baseline triglyceride-glucose (TyG) index, a simple surrogate measure for insulin resistance, is significantly associated with the occurrence of stroke. Nevertheless, the impact of longitudinal patterns of TyG on the stroke risk in hypertensive patients is still unknown. Hence, this study aimed to investigate the association between TyG index trajectory and stroke risk among hypertensive patients.

**Methods:**

This prospective study included 19,924 hypertensive patients from the Kailuan Study who underwent three waves survey and were free of myocardial infarction, cancer and stroke before or during 2010. The TyG index was calculated as ln [fasting triglyceride (mg/dL) × fasting plasma glucose (mg/dL)/2], and latent mixed modelling was used to identify the trajectory of TyG during the exposure period (2006–2010). Furthermore, the Cox proportional hazard models were applied to estimate the hazard ratio (HR) and 95% confidence interval (CI) for incident stroke of different trajectory groups.

**Results:**

Five distinct TyG trajectory were identified during 2006–2010: low-stable (n = 2483; range, 8.03–8.06), moderate low-stable (n = 9666; range, 8.58–8.57), moderate high-stable (n = 5759; range, 9.16–9.09), elevated-stable (n = 1741; range, 9.79–9.75), and elevated-increasing (n = 275; range, 10.38–10.81). During the median follow-up of 9.97 years, 1,519 cases of incident stroke were identified, including 1,351 with ischemic stroke and 215 with hemorrhage stroke. After adjusting for confounding variables, the HR and 95% CI of stroke were 2.21 (1.49,3.28) for the elevated-increasing group, 1.43 (1.13,1.83) for the elevated-stable group, 1.35 (1.10,1.64) for the moderate high-stable group, 1.26 (1.06,1.52) for the moderate low-stable group, respectively, when compare with the low-stable group. Similar results were observed in ischemic stroke, but a significant association was not found between TyG trajectory and risk of hemorrhage stroke.

**Conclusion:**

A long-term elevated TyG index in hypertensive patients is associated with an increased risk of stroke, especially ischemic stroke. This finding implies that regular monitoring of TyG index may assist in identifying individuals at a higher risk of stroke among patients with hypertension.

**Supplementary Information:**

The online version contains supplementary material available at 10.1186/s12933-022-01577-7.

## Background

Stroke is still the primary cause for disability and mortality in view of the 2019 Global Burden of Disease Study [1]. Stroke has more than two million new cases per year and is related to the largest life-year disability-adjusted loss for any disease in China, indicating an increasing and high burden of stroke [2]. Existing evidence has shown that hypertension is a major risk factor for stroke, occupying 2/3 of stroke in developing countries and 1/3 in developed countries [3, 4]. The absolute burden and prevalence of hypertension are increasing worldwide, particularly in low- and middle-income countries [5]. A national survey published in 2018 revealed that the weighted prevalence of hypertension in Chinese adults was 23.2%, equivalent to approximately 244 million patients [6]. Compared to a relatively healthy population, hypertensive patients have a greater likelihood of suffering from a stroke. Therefore, it is essential to identify the population at a high risk of stroke in hypertensive patients and better prevent the occurrence of stroke.

Abnormal glucose and lipid metabolism are very commonplace in individuals with hypertension, while insulin resistance (IR) plays a vital role in this biological process [7–9]. IR has been proven to be largely responsible for endothelial dysfunction and is regarded as a key risk factor for the occurrence and development of stroke [10, 11]. The hyperinsulinemic-euglycemic clamp test is the golden-standard way of assessing IR, whose result is accurate but time-consuming and labor-intensive [12]. Recently, the TyG index has been shown to be a simple and reliable surrogate index for IR, as calculated by fasting triglyceride (TG) and fasting plasma glucose (FPG) evels [13, 14]. It is well established that elevated TyG increases the stroke risk in general population [15, 16], but the report about hypertensive patients is limited. In addition, the TyG index is susceptible to changes caused by many external factors. However, previous studies on the association between stroke risk and TyG index only used a single measure of TyG level and did not explore the longitudinal association between changes in TyG over time and stroke events in patients with hypertension.

Therefore, in the present study, we aim to determine the relationship between the longitudinal patterns of TyG index and stroke risk in hypertensive patients by using TyG trajectory during a 4-year period from the Kailuan study cohort (Registration Number: CHICTR-TNRC-11001489).

## Methods

### Study design and participants

The Kailuan Study is a large community-based, prospective cohort study to assess the risk factors and progression of CVD, conducted in Tangshan City, China. The detailed study design and methodology have been introduced previously [17, 18]. From 2006 to 2007, employees and retirees of the Kailuan Group at the age of 18–98 were recruited to participate in the baseline survey. Then, they underwent a comprehensive biennial health examination and a questionnaire on medication history, lifestyle factors, and demographic characteristics. A total of 57,914 participants who all participated in the first three surveys (2006–2007, 2008–2009 and 2010–2011) were enrolled in this study. Among them, 23,057 patients with hypertension were identified in the baseline survey (2006–2007). In the current study, 388 patients with myocardial infarction and 577 patients with stroke before 2006, 478 patients with myocardial infarction and 1,046 patients with stroke during 2006–2011 were excluded. Moreover, participants with missing TG or FPG data (n = 416), and cancer (n = 228) in or before the third wave survey (2010–2011) were excluded as well. Finally, 19,924 hypertensive participants were included in this study analysis (Additional file 1: Fig. S1). Also, the TyG index trajectory was established over the exposure period (from 2006 to 2010) to predict stroke risk in participants with hypertension from 2010 to 2020. This study was approved by the Ethics Committee of the Kailuan General Hospital and carried out based on the Declaration of Helsinki, with written informed consent provided by all participants.

### Data collection and definition

All participants underwent baseline and follow-up surveys in 11 hospitals in the Kailuan community. A self-report questionnaire by trained staff was used to collect information on demographic characteristics, disease history (hypertension, diabetes, CVD, etc.), medication history (lipid-lowering, hypoglycemic, and antihypertensive drugs, etc.), and lifestyle factors (physical activity, drinking status, and smoking status etc.). A tape rule was used for measuring the height to the nearest 0.1 cm, and a calibrated platform scale was used for measuring weight to the nearest 0.1 kg when participants were shoeless and wore light clothes. Body mass index (BMI) was measured by dividing body weight (kg) by the square of height (m^2^). In addition, blood pressure (BP) was detected at least twice in the right upper arm with a calibrated mercury sphygmomanometer when participants were in a seated posture. If the difference between the two measured values was ≥ 5 mmHg, BP was measured again, and the average results of the three measured values of BP were recorded.

Current-smoker was defined as smoking for over 1 year, smoking on average ≥ 1 cigarette/d, and still smoking in the last year. Current-drinker was defined as drinking for over 1 year, drinking on average ≥ 100 ml/d, and still drinking in the last year. Educational level was divided into junior high school or below, and senior high school or above. Active physical exercise was defined as exercise ≥ 4 times a week, each time duration at least 20 min. Atrial fibrillation was diagnosed according to the following electrocardiograph criteria: (1) irregular R-R intervals; (2) absence of repeating P waves; and (3) irregular atrial activity, in line with the European Society of Cardiology guidelines [19]. Hypertension was defined as systolic blood pressure (SBP) ≥ 140 mmHg, or/and diastolic blood pressure (DBP) ≥ 90 mmHg, or/and taking antihypertensive drugs in the past two weeks [20]. Diabetes mellitus was defined as FPG ≥ 7.0 mmol/L, or/and having a history of clearly diagnosed diabetes or/and taking hypoglycemic drugs [21].

### Biochemical indicators and TyG index

Blood samples were collected from the antecubital vein of participants in the morning after overnight fasting for at least 8 h. Biochemical indicators such as high-sensitivity C-reactive protein (hs-CRP), FPG, TG, low-density lipoprotein cholesterol (LDL-C), high-density lipoprotein cholesterol (HDL-C), and total cholesterol (TC) were tested by examiners using an automatic analyzer (Hitachi 747, Tokyo, Japan) at the central laboratory of hospital. The TyG index was measured ln (fasting TG [mg/dL] × FPG [mg/dL]/2), as previously described [22].

### Assessment of stroke

The primary outcome of this study was the first occurrence of stroke. The subgroup of stroke includes hemorrhagic stroke and ischemic stroke, while hemorrhagic stroke also includes intracerebral hemorrhage and subarachnoid hemorrhage. Assessments of stroke incidence were conducted once a year during the follow-up period, as described previously [23]. To be brief, potential stroke events was ascertained from 4 complementary sources: (1) Municipal Social Insurance Institution, which covered all participants, (2) Hospital Discharge Register centers, (3) death certificates, and (4) questionnaire survey (biennially since 2006). Potential cases of stroke included those who were identified by the ICD-10 (I60 to I61 for hemorrhagic stroke, and I63 for ischemic stroke) [24] based on the first 3 sources or those who self-reported in questionnaire survey. Stroke was diagnosed by brain computed tomography or magnetic resonance imaging scans, combined with neurological symptoms and signs according to the World Health Organization criteria [25]. Additionally, annual discharge records were collected and reviewed by a group of experienced experts to confirm suspected stroke cases. Fatal stroke cases were determined by medical records, autopsy reports, or death certificates with stroke as the underlying cause of death. The cases of nonfatal stroke were classified as the sudden onset of focal neurological deficit with vascular mechanism lasting more than 24 h. All participants were followed up until incident stroke diagnosis, death or December 31, 2020, whichever came first.

### Statistical analysis

All statistical analyses were conducted using the SAS 9.4 (SAS Institute, Inc, Cary, NC) software. The TyG trajectory during 2006 to 2010 in hypertensive patients was identified by latent mixed modelling within the Proc Traj procedure of SAS [26–28]. The Bayesian information criterion (BIC) was adopted to assess the model fit and determine the model with five patterns as the best fit.

For the baseline description, continuous variables with a skewed distribution were considered to be the median with an interquartile range (25–75%), and continuous, normally distributed variables were regarded as the mean ± standard deviation (SD). Number and percentage (%) were used to describe categorical variables. According to distribution, the Kruskal Wallis test or one-way analysis of variance was used for comparisons of continuous variables and the chi-square test was conducted to analyze categorical variables. The incidence density was measured as the number of events divided by the total person-years of follow-up and presented as events per 1000 person-years. Kaplan–Meier method was used to compute stroke and its subgroup cumulative incidence of each trajectory group and the log-rank test was used for comparison. Additionally, the Cox proportional hazard models were established to analyze the HR and 95% CI of stroke for other trajectory groups when compare with the low-stable group and the proportional hazards assumption was satisfied. Model 1 adjusted for sex and age. Model 2 adjusted for sex, age, heart rate, BMI, SBP, DBP, hs-CRP, TC, current-smoker (yes or no), current-drinker (yes or no), active physical activity (yes or no), education level (junior high school or below, and senior high school or above), atrial fibrillation (yes or no) and diabetes mellitus (yes or no). Further adjustments were made for the use of antihypertensive drugs (yes or no), hypoglycemic drugs (yes or no), and lipid-lowering drugs (yes or no) in the model 3. Moreover, subgroup analyses were stratified by sex, age (< 60 years vs. ≥ 60 years), BMI (< 28 kg/m^2^ vs ≥ 28 kg/m^2^) and BP (< 140/90 mmHg vs ≥ 140/90 mmHg).

Several sensitivity analyses were performed to verify the robustness of the results. First, to examine whether this association could be explained by a single TyG during the follow-up, additional adjustments were made for TyG in 2006 and TyG in 2010, respectively. Second, considering the change of covariates during 2006–2010, we additionally conducted a sensitivity analysis of adjusting for the change degree of covariates. Third, outcome events occurring in the first year of follow-up were excluded to minimize potential reverse causality. Fourth, participants with the history of atrial fibrillation were excluded given the impact of atrial fibrillation on the stroke risk. Finally, taking account of the effects of different drug treatments on the results, we also conducted three sensitivity analyses of excluding participants using antihypertensive drugs, hypoglycemic drugs or lipid-lowering drugs, respectively. In this study, statistical significance was indicated as *p* < 0.05 (two-sided test).

## Results

### Baseline characteristics

**Fig. 1:**
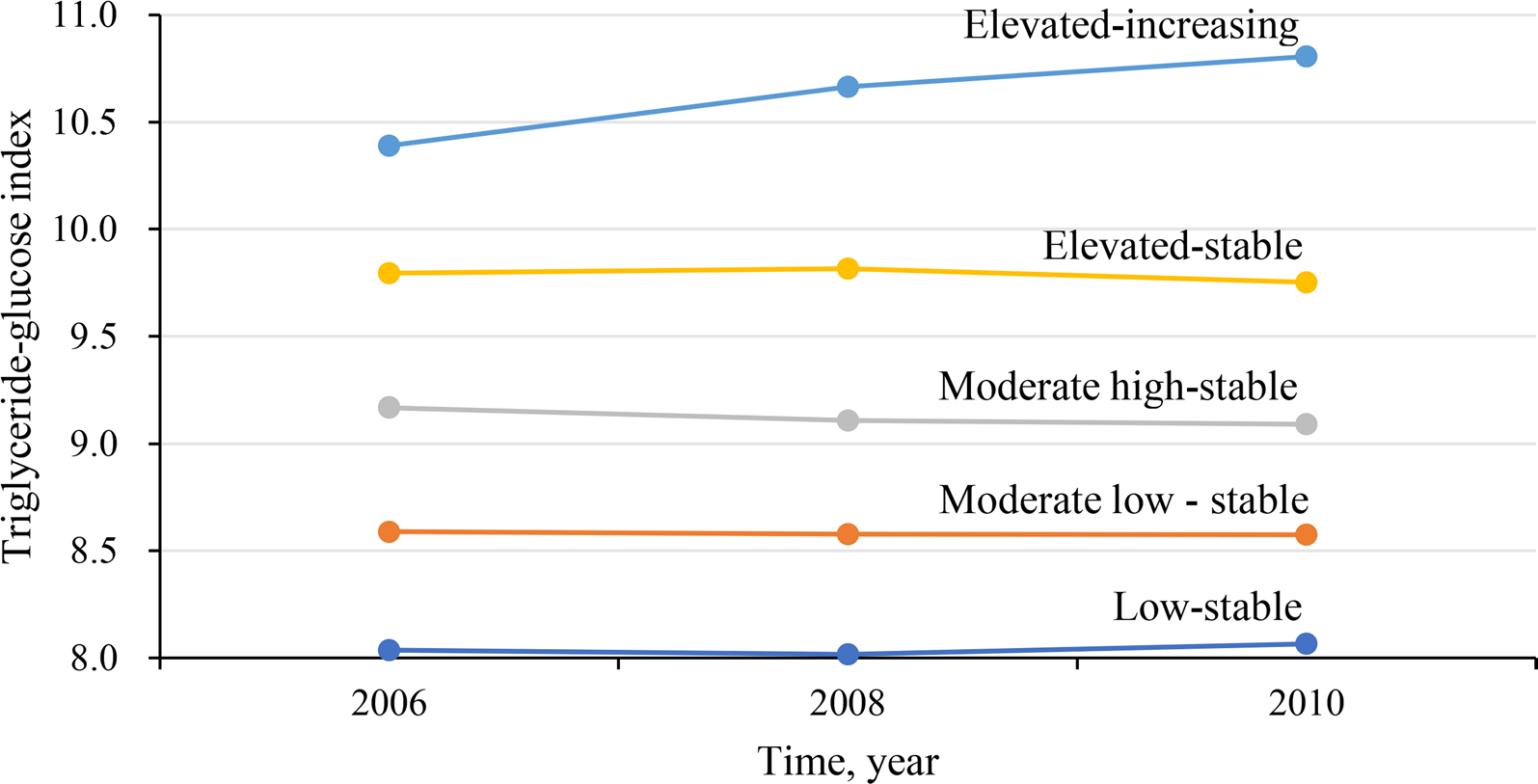
Triglyceride-glucose index trajectory in hypertensive patients during 2006–2010

The baseline characteristics of participants were shown in Table 1. A total of 19,924 hypertensive participants were included in this study, whose mean age at the baseline was 56.78 ± 11.13 years. Of them, 16,342 (82.02%) were men and 3,582 (17.98%) were women. In the present study, five distinct trajectory patterns were identified by latent mixed modelling according to the TyG index change patterns from 2006 to 2010 (Fig. 1): the participants (n = 2483) who maintained low TyG levels (mean TyG range, 8.03–8.06, referred to as the “low-stable” group), the participants (n = 9666) who consistently had moderate-low TyG level (mean TyG range, 8.58–8.57, referred to as the “moderate low-stable” group), the participants (n = 5759) who maintained moderate-high TyG level (mean TyG range, 9.16–9.09, referred to as the “moderate high-stable” group), the participants (n = 1741) who consistently had elevated TyG level (mean TyG range, 9.79–9.75, referred to as the “elevated-stable” group), and the participants (n = 275) who began with elevated and then had a slight increasing TyG level (mean TyG range, 10.38–10.81, referred to as the “elevated-increasing” group). Relative to the low-stable group, participants in the elevated-stable and elevated-increasing group were more likely to be young person, current smokers, current drinkers and had higher heart rate, BMI, SBP, DBP, total cholesterol, and hs-CRP levels, while featured with the higher prevalence of diabetes mellitus and proportion of drugs treatment.

**Table 1:** Baseline characteristics of 19924 hypertensive patients according to the trajectories of TyG index from 2006 to 2010

Variables	Low-Stable	Moderate low-stable	Moderate high-stable	Elevated-stable	Elevated-increasing	P
N	2483	9666	5759	1741	275	–
Age, years	59.38 ± 11.35	57.29 ± 11.35	55.76 ± 10.71	54.49 ± 10.13	51.88 ± 9.29	< 0.01
Male n (%)	2029 (81.72)	7886 (81.58)	4708 (81.75)	1478 (84.89)	241 (87.64)	< 0.01
Heart rate, beats/min	72.72 ± 10.62	73.97 ± 10.91	75.51 ± 11.29	77.89 ± 11.77	78.69 ± 10.54	< 0.01
BMI, kg/m^2^	24.31 ± 3.27	25.61 ± 3.33	26.68 ± 3.31	26.99 ± 3.29	27.01 ± 3.30	< 0.01
SBP, mmHg	140.83 ± 19.51	140.69 ± 19.11	142.21 ± 18.73	143.69 ± 19.16	142.43 ± 17.13	< 0.01
DBP, mmHg	87.89 ± 10.71	88.89 ± 10.71	90.09 ± 10.68	90.93 ± 11.11	91.65 ± 10.38	< 0.01
Total cholesterol, mmol/L	4.67 ± 0.87	4.95 ± 0.92	5.23 ± 1.03	5.43 ± 1.17	6.44 ± 1.79	< 0.01
hs-CRP, mmol/L	1.26 (0.62, 3.01)	1.04 (0.40, 2.60)	1.30 (0.50, 3.10)	1.60 (0.70, 3.70)	1.61 (0.90, 3.72)	< 0.01
TyG in 2006	7.94 ± 0.34	8.55 ± 0.37	9.22 ± 0.44	9.84 ± 0.52	10.45 ± 0.60	< 0.01
TyG in 2008	7.91 ± 0.32	8.56 ± 0.36	9.15 ± 0.42	9.87 ± 0.53	10.67 ± 0.63	< 0.01
TyG in 2010	7.97 ± 0.32	8.53 ± 0.38	9.13 ± 0.44	9.81 ± 0.49	10.86 ± 0.61	< 0.01
Current smoker, n (%)	800 (32.22)	3416 (35.34)	2251 (39.09)	794 (45.61)	136 (49.45)	< 0.01
Current drinker, n (%)	698 (28.11)	2983 (30.86)	2126 (36.92)	761 (43.71)	134 (48.73)	< 0.01
Active physical activity, n (%)	415 (16.71)	1573 (16.27)	865 (15.02)	230 (13.21)	42 (15.27)	< 0.01
Senior high school or above, n (%)	351 (14.14)	1865 (19.29)	1240 (21.53)	376 (21.60)	64 (23.27)	< 0.01
Atrial fibrillation, n (%)	33 (1.33)	90 (0.93)	43 (0.75)	10 (0.57)	0 (0.00)	0.02
Diabetes mellitus, n (%)	47 (1.89)	648 (6.70)	1296 (22.50)	793 (45.55)	176 (64.01)	< 0.01
Hypoglycemic drugs, n (%)	19 (0.77)	231 (2.39)	482 (8.37)	284 (16.31)	65 (23.64)	< 0.01
Anti-hypertensive drugs, n (%)	449 (18.08)	1969 (20.37)	1487 (25.82)	504 (28.95)	103 (37.45)	< 0.01
Lipid-lowering drugs, n (%)	9 (0.36)	82 (0.85)	114 (1.98)	53 (3.04)	13 (4.73)	< 0.01

*TyG* triglyceride-glucose index, *BMI* body mass index, *SBP* systolic blood pressure, *DBP* diastolic blood pressure, *hs-CRP* high-sensitivity C-reactive protein

### Relationship between stroke risk and TyG index trajectory

**Fig. 2:**
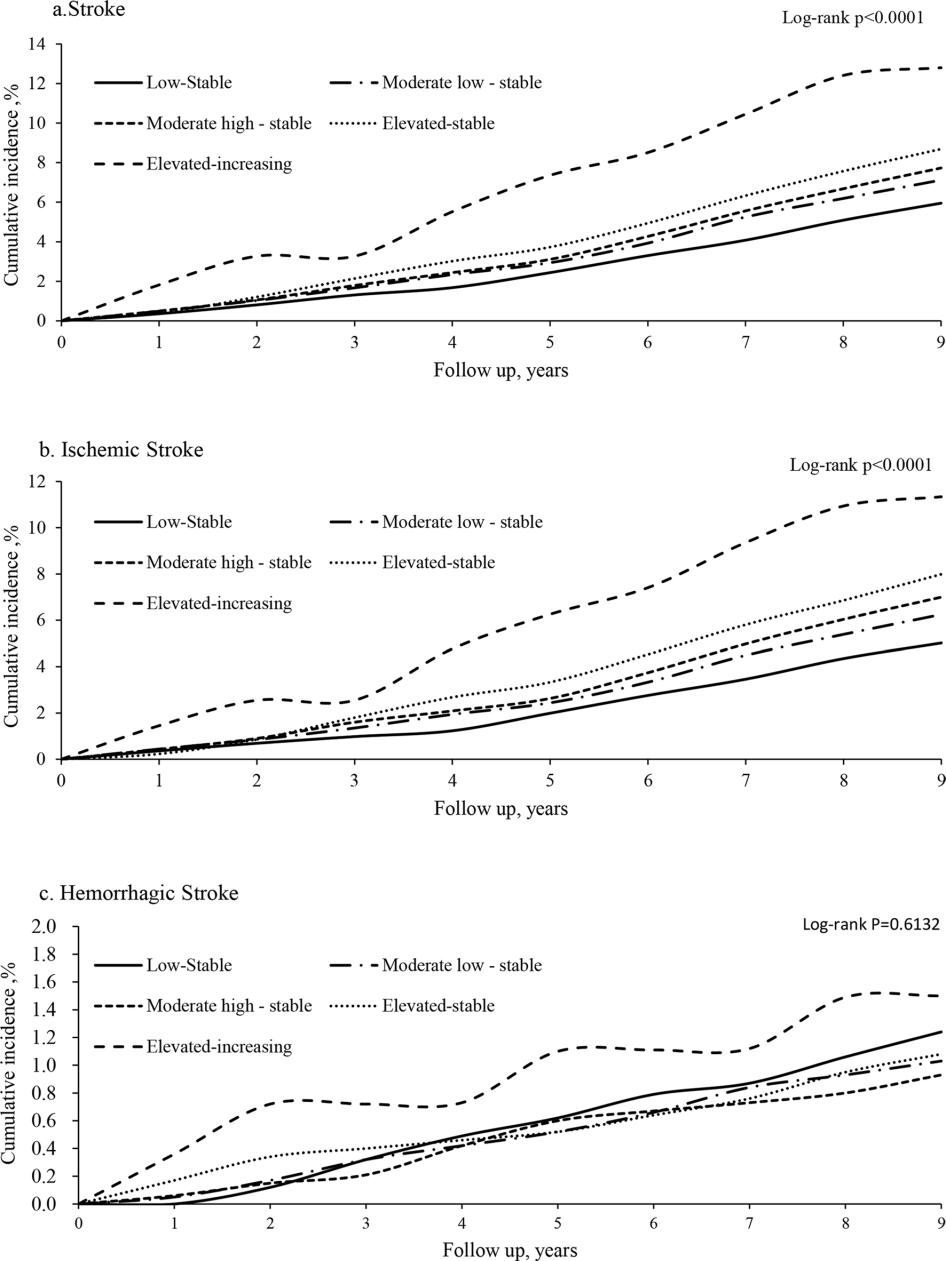
Kaplan–Meier curves of incidence of outcomes according to TyG index trajectory. **a** Stroke; **b** Ischemic stroke; **c** Hemorrhage stroke

After a median follow-up of 9.97 years, 1,519 cases of incident stroke were identified (1,351 with ischemic stroke, and 215 with hemorrhagic stroke). The Kaplan–Meier curve showed that participants in the elevated-increasing TyG group had a higher risk of stroke, ischemic stroke instead of hemorrhagic stroke than those in other trajectory groups (log-rank test, p < 0.0001, Fig. 2a, b; p = 0.6132, Fig. 2c). After adjusting for potential confounders, the HR and 95% CI for stroke, ischemic stroke and hemorrhagic stroke were 2.21 (1.49,3.28), 2.16 (1.42,3.29) and 1.77 (0.58,5.37) in the elevated-increasing group, 1.43 (1.13,1.83), 1.51 (1.16,1.95) and 1.02 (0.55,1.90) in the elevated-stable group, 1.35 (1.10,1.64), 1.40 (1.13,1.73) and 0.86 (0.54,1.38) in the moderate high-stable group, 1.26 (1.06,1.52), 1.30 (1.07,1.57) and 0.94 (0.63,1.40) in the moderate low-stable group, respectively, when compare with the low-stable group (Table 2).

**Table 2:** The hazard ratio (HR) of stroke and its subgroup in hypertensive patients according to trajectories of TyG from 2006 to 2010

	Low-Stable	Moderate low-stable	Moderate high-stable	Elevated-stable	Elevated-increasing
Stroke					
Case/Total	149/2483	715/9666	460/5759	159/1741	36/275
IR	6.52	7.97	8.62	9.91	14.67
Model 1	1 (reference)	1.33 (1.11, 1.58)	1.53 (1.27, 1.85)	1.84 (1.47, 2.31)	2.97 (2.06, 4.29)
Model 2	1 (reference)	1.27 (1.07, 1.53)	1.35 (1.11, 1.64)	1.44 (1.12, 1.84)	2.24 (1.51, 3.32)
Model 3	1 (reference)	1.26 (1.06, 1.52)	1.35 (1.10, 1.64)	1.43 (1.13, 1.83)	2.21 (1.49, 3.28)
Ischemic stroke					
Case/Total	127/2483	628/9666	417/5759	147/1741	32/275
IR	5.54	6.98	7.79	9.13	12.95
Model 1	1 (reference)	1.37 (1.13, 1.66)	1.64 (1.34, 2.01)	2.01 (1.58, 2.55)	3.11 (2.10, 4.60)
Model 2	1 (reference)	1.31 (1.07, 1.58)	1.41 (1.14, 1.74)	1.52 (1.17, 1.97)	2.20 (1.45, 3.34)
Model 3	1 (reference)	1.30 (1.07, 1.57)	1.40 (1.13, 1.73)	1.51 (1.16, 1.95)	2.16 (1.42, 3.29)
Hemorrhagic stroke					
Case/Total	32/2483	106/9666	54/5759	19/1741	4/275
IR	1.37	1.15	0.98	1.14	1.52
Model 1	1 (reference)	0.89 (0.60, 1.32)	0.79 (0.51, 1.24)	0.96 (0.54, 1.70)	1.37 (0.48, 3.89)
Model 2	1 (reference)	0.92 (0.62, 1.39)	0.84 (0.52, 1.36)	0.99 (0.53, 1.86)	1.71 (0.56, 5.19)
Model 3	1 (reference)	0.94 (0.63, 1.40)	0.86 (0.54, 1.38)	1.02 (0.55, 1.90)	1.77 (0.58, 5.37)

*TyG* triglyceride-glucose index, *IR* incidence rate (per 1000 person-years); Model: 1 adjusted for age, sex; Model 2: adjusted for heart rate, BMI, SBP, DBP, total cholesterol, hs-CRP, current smoker, current drinker, physical activity, education level, diabetes mellitus and atrial fibrillation on the basis of model 1; Model 3: included variables in model 2 and further adjusted for hypoglycemic drugs, anti-hypertensive drugs and lipid-lowering drugs

### Subgroup and sensitivity analysis

The results of the subgroup analysis were shown in Table 3, and we did not find significant interaction between the TyG trajectories and age, sex, BMI or BP. In the sensitivity analyses, there was no substantial change in the relationship between TyG index trajectory and stroke risk after additional adjustment for the TyG index in 2006 or 2010 (Additional file 2: Table S1, S2). In addition, the results of adjusting for the change degree of covariates, excluding outcome events that occurred in the first year of follow-up (n = 97) or excluding participants with history of atrial fibrillation (n = 176) was still consistent with the main results (Additional file 2: Table S3–S5). Furthermore, after excluding participants who receiving treatment with the use of lipid-lowering (n = 271), hypoglycemic (n = 1081) or anti-hypertensive drugs (n = 4512), respectively, the result still was steady (Additional file 2: Table S6).

**Table 3:** Subgroup analyses: the hazard ratio (HR) of stroke in hypertensive patients according to trajectories of TyG From 2006 to 2010

	Low-Stable	Moderate low-stable	Moderate high-stable	Elevated-stable	Elevated-increasing	P for interaction
Sex						0.06
Male						
Case/Total	125/2029	632/7886	402/4708	134/1478	31/241	
	1 (reference)	1.37 (1.13, 1.67)	1.46 (1.18, 1.81)	1.47 (1.12, 1.92)	2.25 (1.46, 3.44)	
Female						
Case/Total	24/454	83/1780	58/1051	25/263	5/34	
	1 (reference)	0.77 (0.48, 1.23)	0.81 (0.48, 1.35)	1.26 (0.67, 2.36)	2.28 (0.82, 6.33)	
Age						0.80
Age < 60 y						
Case/Total	57/1346	359/5897	267/3860	103/1278	27/232	
	1 (reference)	1.38 (1.04, 1.83)	1.40 (1.05, 1.91)	1.42 (1.00, 1.98)	2.01 (1.23,3 .29)	
Age ≥ 60 y						
Case/Total	92/1137	356/3769	193/1899	56/463	9/43	
	1 (reference)	1.21 (0.96, 1.53)	1.34 (1.02, 1.76)	1.65 (1.14, 2.38)	3.25 (1.58, 6.70)	
BMI						0.15
BMI < 28						
Case/Total	128/2187	554/7604	305/3949	118/1148	19/174	
	1 (reference)	1.29 (1.06, 1.57)	1.35 (1.08, 1.68)	1.65 (1.26, 2.17)	2.02 (1.20, 3.33)	
BMI ≥ 28						
Case/Total	21/296	161/2062	155/1810	41/593	17/101	
	1 (reference)	1.12 (0.70, 1.80)	1.21 (0.75, 1.95)	0.95 (0.54, 1.67)	2.47 (1.23, 4.97)	
BP						0.83
BP < 140/90 mmHg						
Case/Total	39/951	178/3484	109/1784	34/517	6/71	
	1 (reference)	1.21 (0.85, 1.72)	1.34 (0.90, 1.98)	1.28 (0.77,2.13)	1.64 (0.65, 4.07)	
BP ≥ 140/90 mmHg						
Case/Total	110/1532	537/6182	351/3975	125/1224	30/204	
	1 (reference)	1.27 (1.03, 1.57)	1.32 (1.05, 1.65)	1.47 (1.11, 1.94)	2.42 (1.56, 3.75)	

*TyG* triglyceride-glucose index; Adjusted for age, sex, heart rate, BMI, SBP, DBP, total cholesterol, hs-CRP, current smoker, current drinker, physical activity, education level, diabetes mellitus, atrial fibrillation, hypoglycemic drugs, anti-hypertensive drugs and lipid-lowering drugs.

## Discussion

Five different TyG trajectory patterns for 4 years were identified in this prospective study of 19,924 participants with hypertension from the Kailuan study. Hypertensive patients with a long-term high TyG level, as compared with those in the low-stable group, had a higher risk of stroke and ischemic stroke during the follow-up of 10 years, independent of the baseline TyG. Nevertheless, no significant association was observed between TyG index trajectory and hemorrhagic stroke. Similar results were also found in the subgroup and sensitivity analyses.

The relationship between TyG index and incidence of stroke has been widely investigated in the general population. A cross-sectional study from the rural areas of northeast China indicated that the elevated TyG level is an independent risk factor for stroke and confirmed the TyG value of optimizing the risk stratification of ischemic stroke [15]. Zhao et al. performed a cohort study of 11,777 participants, showing that participants with the highest TyG index had a significantly increased risk of ischemic stroke after a median follow-up of 6 years [29]. Another national observational cohort study from the Korean population also showed that a high TyG index had a strong association with higher subsequent stroke risk [30]. However, most of the studies were conducted in relatively healthy population and very few studies have explored the relationship between TyG index and stroke incidence in hypertensive patients at a high risk of stroke. To our knowledge, only in a cohort study among elderly patients with hypertension, Hu et al. found a positive association between TyG index and the risk of first stroke [31]. Furthermore, previous studies in the general population or hypertensive patients only focused on a single TyG measurement without considering the long-term effect of TyG changes over time on stroke. In this study, trajectories were used to present the TyG change patterns over 4 years to observe its long-term effect on the risk of stroke among hypertensive patients. Our baseline results are consistent with previous findings in patients with hypertension [31, 32], showing that the highest TyG was in the youngest patients with hypertension. Moreover, our previous work has shown that TG level in the young people is generally higher than in the older, which was also found in the other studies [33–35]. After adjusting for potential confounding factors, our study extended the current database by indicating that hypertensive patients with a long-term elevated TyG level had a higher risk of stroke and ischemic stroke, relative to those with a low-stable TyG index. Moreover, the association was not attenuated through additionally adjusting for baseline TyG level. Therefore, routine monitoring of the TyG index may be helpful for identifying people at higher risk of stroke in hypertensive patients, providing new evidence for the prevention of stroke in clinical practice.

However, no association was observed between the TyG index trajectory and hemorrhagic stroke, a subtype of stroke with relatively low incidence. In this study, patients with hypertension in the elevated-increasing group have no significant increased risk of hemorrhagic stroke. Wang et al. showed that the baseline and long-term cumulative mean TyG index could predict the risk of stroke and ischemic stroke instead of intracerebral hemorrhage in the general population [16]. Additionally, a meta-analysis of 11 studies also revealed that the TyG index had no association with hemorrhagic stroke, indirectly supporting our results in hypertensive patients [36]. It is noteworthy that hypertension is an independent risk factor for hemorrhagic stroke [37], but a meta-analysis reported that hyperlipidemia may provide the effect of preventing vascular bleeding and the hemorrhagic stroke risk decreases by 7% per 1 mmol/L TG increase [38]. Therefore, the combined effect of the two opposite effects may cause non-significant statistical relevance in the TyG index trajectory and hemorrhagic stroke among hypertensive patients.

The mechanisms underlying the relationship between long-term change of TyG index and the risk of stroke and ischemic stroke remain unclear, but they may be related to IR. First, IR has associations with chronic inflammation, coagulation dysfunction, and oxidative stress, which may lead to the occurrence of hypertension, diabetes, and hyperlipidemia [39–41]. In our study, hypertensive patients in the elevated-increasing group also have a higher prevalence of diabetes mellitus and hyperlipidemia, increasing the risk of stroke jointly. Second, arterial stiffness is a key component of the pathogenesis of stroke [42], and IR plays a key role in endothelial dysfunction and enhances the atherosclerotic process [43]. Previous studies have indicated that the elevated TyG level has a significant association with arterial stiffness in the population with hypertension [32, 44]. At last, participants with a long-term high TyG level have higher BMI, heart rate, SBP, DBP, and TC levels, and tend to be current smokers or drinkers, which are the common risk factor for stroke. Thus, it may lead to a significantly increased risk of stroke.

The strengths of the present study include that it is the first large prospective study to investigate the association of longitudinal pattern of TyG index and stroke risk among patients with hypertension. The trajectories were used to represent changes in TyG level over time in hypertensive patients and the possible confounders affecting the risk of stroke were adjusted in the analysis, which ensures the reliability of the outcomes to some extent. However, several limitations should be noted in this study. First, the homoeostasis model assessment of IR (HOMA-IR) or the hyperinsulinemic-euglycemic clamp test of IR was not conducted on participants, but these methods of assessment are complex and expensive, thus, it is not suitable for routine monitoring. Furthermore, the TyG index have been proven to be great associate with insulin clamp test and HOMA-IR, as a simple surrogate index [45, 46]. Second, the causal relationship between the TyG index trajectory and stroke risk in hypertensive patients cannot be demonstrated because it was an observational study. Third, the diagnosis of diabetes was based on a single measurement of FPG but not oral glucose tolerance test (OGTT) and hemoglobin A1c (HbA1c) level might underestimate the diabetes incidence. Furthermore, the hypoglycemic drugs in this study specifically refer to oral hypoglycemic drugs, and we did not collect the information of insulin or glucose-lowering treatments plus dietary control, which may have a slight effect on the results. Finally, the participants in this study were restricted to northern Chinese adults who live in the Kailuan community, and fewer of them are women, thus, the generalizability is relatively limited. Further studies with multiple regions, ethnicities, populations, particularly those with a balanced proportion of men and women, are still warranted to validate our results.

## Conclusions

In conclusion, a long-term elevated TyG index in hypertensive patients is associated with an increased risk of stroke and ischemic stroke, but no significant association was found between TyG index trajectory and risk of hemorrhagic stroke. These findings highlight the importance of monitoring longitudinal TyG index patterns and early controlling glucose and lipid to prevent the occurrence of stroke among patients with hypertension.

## Supplementary Information

**Additional file 1: Fig. S1.** Flow chart of inclusion and exclusion. The flowchart of 19,924 hypertensive patients included in the final analyses.

**Additional file 2: Table S1.** Sensitivity Analysis of additionally adjusted for TyG in 2006. **Table S2.** Sensitivity Analysis of additionally adjusted for TyG in 2010. **Table S3.** Sensitivity Analysis of adjusted for the change degree of covariates from 2006 to 2010. **Table S4.** Sensitivity Analysis of excluding Outcome Events within the first year of follow-up. **Table S5.** Sensitivity Analysis of excluding participants with the history of atrial fibrillation. **Table S6.** Sensitivity Analysis of excluding participants with the use of anti-hypertensive drugs, hypoglycemic drugs or lipid-lowering drugs, respectively.

## Data Availability

The datasets used and analyzed during the current study are available from the corresponding author on reasonable request.

## Abbreviations

TyG index: Triglyceride-glucose index
IR: Insulin resistance
CVD: Cardiovascular disease
TG: Triglyceride
FPG: Fasting plasma glucose
BMI: Body mass index
BP: Blood pressure
SBP: Systolic blood pressure
DBP: Diastolic blood pressure
TC: Total cholesterol
HDL-C: High-density lipoprotein-cholesterol
hsCRP: High-sensitivity C-reactive protein
LDL-C: Low-density lipoprotein-cholesterol
HR: Hazard ratio
CI: Confidence interval
