# Supplementary material for: Triglyceride-glucose index trajectory and stroke incidence in patients with hypertension: a prospective cohort study
Source: Cardiovasc Diabetol. 2022 Jul 27;21:141. doi: 10.1186/s12933-022-01577-7 (PMC9331781; doi:10.1186/s12933-022-01577-7)

**Additional file 1**

**Additional figure**

**Fig. S1.** Flow chart of inclusion and exclusion.

**Caption:** The flowchart of 19,924 hypertensive patients included in the final analyses.


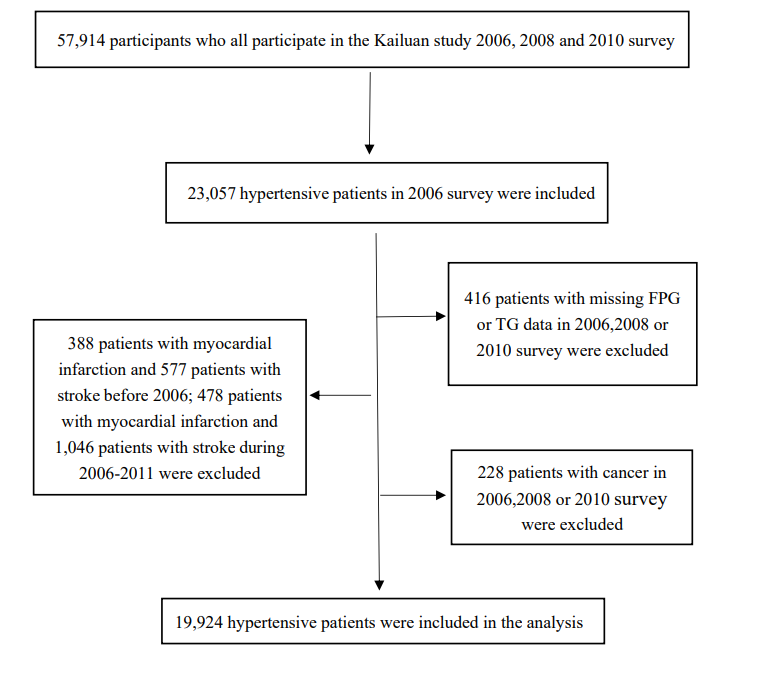

Supplement: Supplementary file 1 — Additional file 1: Fig. S1. Flow chart of inclusion and exclusion. The flowchart of 19,924 hypertensive patients included in the final analyses. [file 12933_2022_1577_MOESM1_ESM.docx]
