# Supplementary material for: Triglyceride-glucose index trajectory and stroke incidence in patients with hypertension: a prospective cohort study
Source: Cardiovasc Diabetol. 2022 Jul 27;21:141. doi: 10.1186/s12933-022-01577-7 (PMC9331781; doi:10.1186/s12933-022-01577-7)
Supplement: Supplementary file 2 — Additional file 2: Table S1. Sensitivity Analysis of additionally adjusted for TyG in 2006. Table S2. Sensitivity Analysis of additionally adjusted for TyG in 2010. Table S3. Sensitivity Analysis of adjusted for the change degree of covariates from 2006 to 2010. Table S4. Sensitivity Analysis of excluding Outcome Events within the first year of follow-up. Table S5. Sensitivity Analysis of excluding participants with the history of atrial fibrillation. Table S6. Sensitivity Analysis of excluding participants with the use of anti-hypertensive drugs, hypoglycemic drugs or lipid-lowering drugs, respectively. [file 12933_2022_1577_MOESM2_ESM.docx]

**Additional file 2**

**Additional tables**

**Table S1.** Sensitivity Analysis of additionally adjusted for TyG in 2006

**Table S2.** Sensitivity Analysis of additionally adjusted for TyG in 2010

**Table S3.** Sensitivity Analysis of adjusted for the change degree of covariates from 2006 to 2010

**Table S4.** Sensitivity Analysis of excluding Outcome Events within the first year of follow-up

**Table S5.** Sensitivity Analysis of excluding participants with the history of atrial fibrillation

**Table S6.** Sensitivity Analysis of excluding participants with the use of anti-hypertensive drugs, hypoglycemic drugs or lipid-lowering drugs, respectively

**Table S1.** Sensitivity Analysis of additionally adjusted for TyG in 2006

|  | Low-Stable | Moderate low-stable | Moderate high-stable | Elevated-stable | Elevated-increasing |
| --- | --- | --- | --- | --- | --- |
| Case/Total | 149/2483 | 715/9666 | 460/5759 | 159/1741 | 36/275 |
| IR | 6.52 | 7.97 | 8.62 | 9.91 | 14.67 |
|  | 1 (Ref.) | 1.27 (1.04,1.54) | 1.33 (1.03,1.71) | 1.41 (1.01,1.98) | 2.16 (1.31,3.57) |

Note: TyG, triglyceride-glucose index, IR，incidence rate (per 1000 person-years); Adjusted for age, sex, heart rate, BMI, SBP, DBP, total cholesterol, hs-CRP, current smoker, current drinker, physical activity, education level, diabetes mellitus, atrial fibrillation, hypoglycemic drugs, anti-hypertensive drugs, lipid-lowering drugs and TyG in 2006.

**Table S2.** Sensitivity Analysis of additionally adjusted for TyG in 2010

|  | Low-Stable | Moderate low-stable | Moderate high-stable | Elevated-stable | Elevated-increasing |
| --- | --- | --- | --- | --- | --- |
| Case/Total | 149/2483 | 715/9666 | 460/5759 | 159/1741 | 36/275 |
| IR | 6.52 | 7.97 | 8.62 | 9.91 | 14.67 |
|  | 1 (Ref.) | 1.19 (1.00,1.46) | 1.20 (0.94,1.52) | 1.22 (0.86,1.66) | 1.66 (1.01,2.79) |

Note: TyG, triglyceride-glucose index, IR，incidence rate (per 1000 person-years); Adjusted for age, sex, heart rate, BMI, SBP, DBP, total cholesterol, hs-CRP, current smoker, current drinker, physical activity, education level, diabetes mellitus, atrial fibrillation, hypoglycemic drugs, anti-hypertensive drugs, lipid-lowering drugs and TyG in 2010.

**Table S3.** Sensitivity Analysis of adjusted for the change degree of covariates from 2006 to 2010

|  | Low-Stable | Moderate low-stable | Moderate high-stable | Elevated-stable | Elevated-increasing |
| --- | --- | --- | --- | --- | --- |
| Case/Total | 149/2483 | 715/9666 | 460/5759 | 159/1741 | 36/275 |
| IR | 6.52 | 7.97 | 8.62 | 9.91 | 14.67 |
|  | 1(Ref.) | 1.35 (1.11,1.64) | 1.51 (1.23,1.86) | 1.65 (1.28,2.14) | 2.73 (1.83,4.08) |

Note: TyG, triglyceride-glucose index, IR，incidence rate (per 1000 person-years); Adjusted for age, sex, heart rate, BMI, SBP, DBP, total cholesterol, hs-CRP, current smoker, current drinker, physical activity, education level, diabetes mellitus, atrial fibrillation, hypoglycemic drugs, anti-hypertensive drugs, and lipid-lowering drugs.

**Table S4.** Sensitivity Analysis of excluding Outcome Events within the first year of follow-up (n=19,827)

|  | Low-Stable | Moderate low-stable | Moderate high-stable | Elevated-stable | Elevated-increasing |
| --- | --- | --- | --- | --- | --- |
| Case/Total | 141/2475 | 667/9618 | 431/5730 | 152/1734 | 31/270 |
| IR | 6.17 | 7.44 | 8.08 | 9.47 | 12.62 |
|  | 1(Ref.) | 1.25 (1.04,1.51) | 1.33 (1.08,1.62) | 1.44 (1.12,1.85) | 2.03 (1.34,3.08) |

Note: TyG, triglyceride-glucose index, IR，incidence rate (per 1000 person-years); Adjusted for age, sex, heart rate, BMI, SBP, DBP, total cholesterol, hs-CRP, current smoker, current drinker, physical activity, education level, diabetes mellitus, atrial fibrillation, hypoglycemic drugs, anti-hypertensive drugs, and lipid-lowering drugs.

**Table S5.** Sensitivity Analysis of excluding participants with the history of atrial fibrillation (n=19,748)

|  | Low-Stable | Moderate low-stable | Moderate high-stable | Elevated-stable | Elevated-increasing |
| --- | --- | --- | --- | --- | --- |
| Case/Total | 147/2450 | 707/9576 | 454/5716 | 159/1731 | 36/275 |
| IR | 6.50 | 7.95 | 8.56 | 9.96 | 14.67 |
|  | 1(Ref.) | 1.27 (1.06,1.52) | 1.33 (1.09,1.62) | 1.44 (1.12,1.84) | 2.19 (1.47,3.24) |

Note: TyG, triglyceride-glucose index, IR，incidence rate (per 1000 person-years); Adjusted for age, sex, heart rate, BMI, SBP, DBP, total cholesterol, hs-CRP, current smoker, current drinker, physical activity, education level, diabetes mellitus, hypoglycemic drugs, anti-hypertensive drugs, and lipid-lowering drugs.

**Table S6.** Sensitivity Analysis of excluding participants with the use of anti-hypertensive drugs, hypoglycemic drugs or lipid-lowering drugs, respectively

|  | Low-Stable | Moderate low-stable | Moderate high-stable | Elevated-stable | Elevated-increasing |
| --- | --- | --- | --- | --- | --- |
| **Excluding participants with the use of Anti-hypertensive drugs** (n=4,512) | | | | | |
| Case/Total | 115/2034 | 522/7697 | 330/4272 | 102/1237 | 20/172 |
| IR | 6.13 | 7.26 | 8.29 | 8.87 | 12.59 |
|  | 1 (Ref.) | 1.26 (1.02,1.54) | 1.42 (1.13,1.78) | 1.44 (1.07,1.91) | 2.14 (1.29,3.56) |
| **Excluding participants with the use of Hypoglycemic drugs** (n=1,081) | | | | | |
| Case/Total | 147/2464 | 694/9435 | 410/5227 | 124/1457 | 24/210 |
| IR | 6.48 | 7.92 | 8.35 | 9.17 | 12.63 |
|  | 1 (Ref.) | 1.28 (1.06,1.53) | 1.34 (1.10,1.64) | 1.38 (1.06,1.79) | 1.99 (1.25,3.15) |
| **Excluding participants with the use of Lipid-lowering drugs** (n=271) | | | | | |
| Case/Total | 149/2474 | 711/9584 | 451/5645 | 156/1688 | 36/262 |
| IR | 6.55 | 8.00 | 8.63 | 10.04 | 15.41 |
|  | 1 (Ref.) | 1.27 (1.06,1.52) | 1.33 (1.09,1.62) | 1.44 (1.12,1.84) | 2.28 (1.54,3.38) |

Note: TyG, triglyceride-glucose index, IR，incidence rate (per 1000 person-years); Adjusted for age, sex, heart rate, BMI, SBP, DBP, total cholesterol, hs-CRP, current smoker, current drinker, physical activity, education level, diabetes mellitus, atrial fibrillation, hypoglycemic drugs, anti-hypertensive drugs, and lipid-lowering drugs.
